# Electrocardiographic Clues for Early Diagnosis of Ventricular Pre-Excitation and Non-Invasive Risk Stratification in Athletes: A Practical Guide for Sports Cardiologists

**DOI:** 10.3390/jcdd11100324

**Published:** 2024-10-14

**Authors:** Simone Ungaro, Francesca Graziano, Sergei Bondarev, Matteo Pizzolato, Domenico Corrado, Alessandro Zorzi

**Affiliations:** Department of Cardiac, Thoracic, Vascular Sciences and Public Health, University of Padova, Via Giustiniani 2, 35128 Padova, Italy; simone.ungaro@studenti.unipd.it (S.U.); francesca.graziano@unipd.it (F.G.); sergei.bondarev@unipd.it (S.B.); matteopizzo92@hotmail.it (M.P.); domenico.corrado@unipd.it (D.C.)

**Keywords:** Wolff–Parkinson–White, ventricular pre-excitation, sport cardiology, sudden cardiac death, athlete’s heart

## Abstract

Ventricular pre-excitation (VP) is a cardiac disorder characterized by the presence of an accessory pathway (AP) that bypasses the atrioventricular node (AVN), which, although often asymptomatic, exposes individuals to an increased risk of re-entrant supraventricular tachycardias and sudden cardiac death (SCD) due to rapid atrial fibrillation (AF) conduction. This condition is particularly significant in sports cardiology, where preparticipation ECG screening is routinely performed on athletes. Professional athletes, given their elevated risk of developing malignant arrhythmias, require careful assessment. Early identification of VP and proper risk stratification are crucial for determining the most appropriate management strategy and ensuring the safety of these individuals during competitive sports. Non-invasive tools, such as resting electrocardiograms (ECGs), ambulatory ECG monitoring, and exercise stress tests, are commonly employed, although their interpretation can sometimes be challenging. This review aims to provide practical tips and electrocardiographic clues for detecting VP beyond the classical triad (short PR interval, delta wave, and prolonged QRS interval) and offers guidance on non-invasive risk stratification. Although the diagnostic gold standard remains invasive electrophysiological study, appropriate interpretation of the ECG can help limit unnecessary referrals for young, often asymptomatic, athletes.

## 1. Introduction

Ventricular pre-excitation (VP) is a disorder caused by a specific disturbance of the electrical system of the heart involving an accessory pathway (AP) able to conduct the electrical impulse between the atria and the ventricles, thus bypassing the atrioventricular node (AVN). Although often asymptomatic, the presence of an AP exposing the patient to an increased risk of recurrent supraventricular and ventricular tachyarrhythmias [1]. When the ECG features are associated with clinical symptoms, it is commonly referred to as Wolff–Parkinson–White syndrome (WPWS) or other pre-excitation syndromes. The typical VP pattern visible at the resting electrocardiogram (ECG) during sinus rhythm can be summarized into three main characteristics: (1) PR interval < 120 ms; (2) presence of a delta wave (slow upstroke or downstroke of the initial part of the QRS complex); and (3) QRS complex duration > 120 ms [2]. More than half of people with VP develop WPWS, which can include palpitations, shortness of breath, dizziness, or even syncope, while cardiac arrest represents the most menacing occurrence [3]. The mechanism involves the onset of atrial fibrillation (AFib), often resulting from degeneration of re-entrant supraventricular tachycardia, which is rapidly conducted to the ventricles through the AP and turns into ventricular fibrillation (VF) [3,4]. For this reason, the ability of an AP to allow a rapid atrioventricular conduction (or, in other words, a low refractoriness) is the main property that is evaluated for risk stratification.

The etiopathogenesis of VP is mainly congenital and, in rare cases, inherited and caused by a PRKAG2 gene mutation, which is associated with left ventricular (LV) hypertrophy and other multisystem diseases [5,6].

Today it is known that APs can be found in more than 1 over 300 young individuals [7], including professional and amateur athletes which, if not properly screened, are exposed to an increased risk of arrhythmic events and sudden cardiac death (SCD). In athletes, VP accounts for at least 1% of SCD, although this percentage could be even larger when considering autopsy-negative cases with sudden unexplained death [8]. Accordingly, VP poses a significant challenge in sports medicine and cardiology, as athletes undergoing preparticipation ECG screening may present with this asymptomatic condition, yet remain at risk for malignant arrhythmic events, including sudden cardiac death. The early identification of an AP and proper risk stratification are crucial to ensure the safe participation of these individuals in competitive sports. While the gold standard for diagnosis remains invasive electrophysiological study, sports cardiologists and physicians often rely on non-invasive tools, such as resting ECG, ambulatory ECG, and exercise stress testing. Although these tools are simple, they can be extremely valuable in clarifying doubts and helping avoid unnecessary invasive procedures, especially in asymptomatic individuals.

This manuscript is specifically designed to aid cardiologists and sports medicine practitioners by reviewing key electrocardiographic features of VP, discussing the different types of AP and their pathophysiological implications, and providing practical tips and electrocardiographic clues to aid in the diagnosis of VP within the sports cardiology context. In particular, more features beyond the three classical ECG signs—shorter PR interval, delta wave, and prolonged QRS interval—are discussed, e.g., ST depression during exercise, QRS modification during sleep, or vagal stimulation. Finally, it explores strategies for non-invasive risk stratification, while emphasizing the importance of limiting invasive assessments whenever possible, particularly in young athletes.

## 2. Electrocardiographic Features

**Figure 1:**
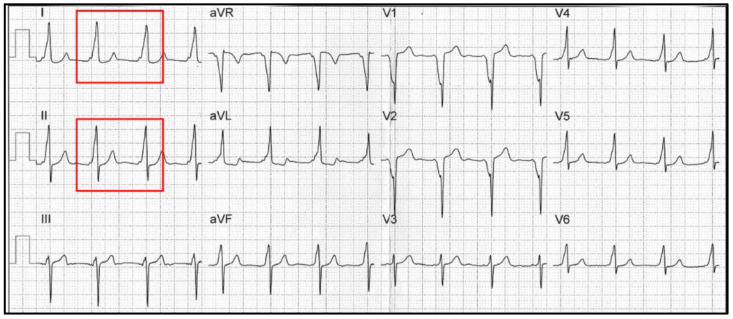
Example of overt ventricular VP. Focus on the delta wave with a short PR interval in DI-DII-V5 (red boxes).

In VP, there are three main electrocardiographic mainstays involving the PR interval and the QRS complex: (1) a PR interval < 120 ms; (2) a delta wave over the QRS complex; and (3) a slurred QRS complex > 120 ms (Figure 1) [2]. Due to the “electrical bypass” of the AVN by the cardiac impulse, the pre-excited PR interval is always shorter than the normal one, although sometimes it could be normal [9].

AP bypasses normal heart conduction system through the AV groove, running through atrial and ventricular myocardium in all the sectors of a transverse plan, except for the regions interspersed by fibrous trigons or other structure of the AV fibrous heart “skeleton” [10]. The transverse plan at the AV valve level can be divided into two parts, one for the mitral valve and one for the tricuspid valve, and each part is further divided into three (mitral valve) or four (tricuspid valve) sectors, each with a different incidence of distribution of APs [11,12,13]. The most common AP location is the free wall of the LV followed by the posterior interventricular septum. The ECG features and their presence in the twelve leads can be used to identify the AP site localization with sufficient accuracy [14,15,16,17].

**Figure 2:**
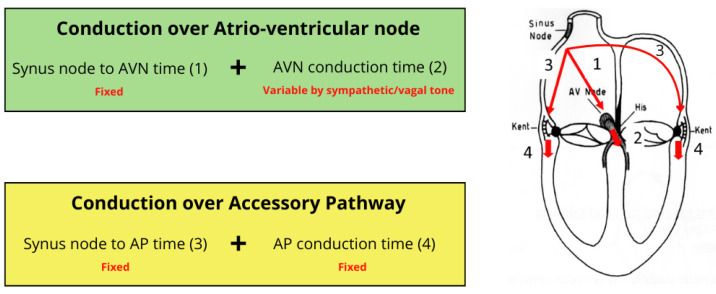
Different conduction times over the atrioventricular node compared with the VP accessory pathway. AVN, atrioventricular node; AP, accessory pathway.

However, it must be considered that the same AP can be more or less visible on the ECG, not only depending on its location, but also on the amount of myocardium that is activated through the AP vs. the normal conduction system, which is a dynamic factor that can change in the same patient [17,18]. This is explained by the different conduction time between the onset of the P wave and ventricular depolarization on the type of pathway. AP conduction time, indeed, is almost stable and “locked” by the sum of the impulse time between the atrial sinus node (ASN) and the AP and through the AP. Conversely, in the physiological condition, the conduction time through the normal conduction system is flexible and regulated by sympathetic vs. vagal tone (Figure 2) [1,3,19].

Later, we discuss how to make an AP more visible on the ECG by increasing the AVN conduction time and thus favoring conduction through the AP.

## 3. Typical vs. Atypical VP Pathways

**Figure 3:**
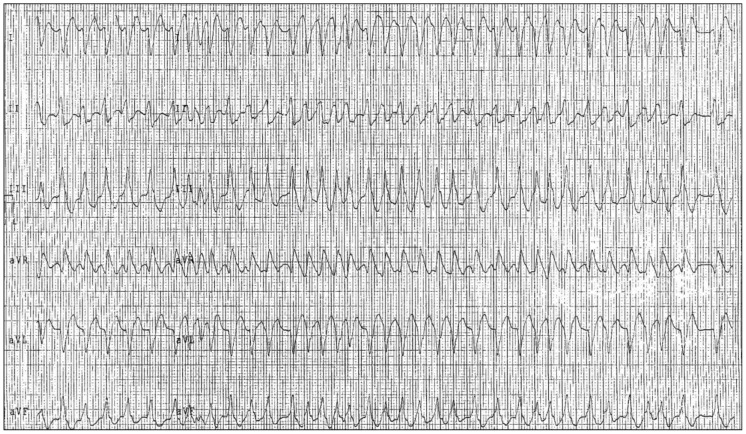
High ventricular rate atrial fibrillation in the presence of Wolff–Parkinson–White syndrome.

Different types of AP exist, either from the perspective of molecular pathogenesis or pathophysiology and location (atrial-fascicular, node-fascicular, fascicular-ventricular) [1,11]. Most APs generate a fast action potential due to the rapid inward sodium current, similar to normal His-Purkinje tissue and atrial or ventricular myocardium. Consequently, they maintain consistent antegrade and retrograde conduction at all rates until the refractory period is reached, at which point conduction is completely blocked (non-decremental conduction). In contrast, the AVN relies on the slow inward calcium current for generating and propagating its action potential, exhibiting decremental conduction. This means that the conduction time of impulses through the AVN increases as the cycle length shortens (heart rate increases) [19]. Therefore, atrioventricular conduction is faster through the AP than through the AVN, a difference that becomes more pronounced at higher heart rates [1,15]. The progressive prolongation of AV nodal conduction time at faster atrial rates serves a protective role, limiting the ventricular response to rapid atrial rates seen in AFib or atrial flutter. This slowing of conduction until only some impulses are transmitted through the AV nodal tissue is known as decremental conduction. In contrast, traditional APs (Kent bundles) depend on the rapid inward sodium current for depolarization and do not exhibit this decremental conduction. As a result, arrhythmias triggered by accessory pathways can conduct frequently and rapidly, potentially leading to very fast ventricular rates during AFib (Figure 3), which may evolve into VF [1,4].

In rare cases, atrial-ventricular AP could show decremental conduction in the antegrade direction. This exposes a diagnostic challenge because, in these cases, VP is not evident during sinus rhythm due to the faster AVN conduction than AP conduction. These cases are frequently seen when the AP originates from the postero-septal sector of mitral/tricuspid annulus [20].

Several AP types exist, each with its own features, origin, and localization [1,11,15]. An important distinction must be made between typical vs. atypical VP APs.

Typically, the classic pathway is represented by the bundle of Kent (atrioventricular bypassing bundle), which is the substrate of the Wolff–Parkinson–White syndrome [15]. This kind of pathway bypasses the AV node and the His-Purkinje fibers, connecting the atrial myocardium directly with the ventricular myocardium fibers. Atypical pathways connect the atrial myocardium to other cardiac conduction structures, such as the AVN or His bundle, or directly to Purkinje fibers.

When the atrium is directly connected to the central or distal AVN, through the so-called “James fibers” [21,22], AV nodal conduction is enhanced, potentially causing Lown–Ganong–Levine syndrome [23,24]. James fibers are also characterized by a short PR interval without showing a delta wave at the baseline ECG [20,21].

Other than Atrio-Hisian tracts connecting the atrial myocardium to the His bundle, various types of Hisian-fascicular pathways exist. These tracts are also known as “Mahaim fibers”, and are bridged between the atria, AVN, and His bundle, to distal Purkinje fibers, or, less frequently, to the ventricular myocardium, generating atrio-fascicular, node-fascicular, and fasciculo-fascicular pathways (often benign) [22,25,26]. These kinds of AP are generally observed in healthy hearts and have excellent prognosis. Unlike Kent fibers, Mahaim fibers are decremental and node-like, and thus are not able to conduct AFib at a high frequency to the ventricles [26]. These differences are crucial for the diagnosis and treatment of the respective arrhythmias associated with each pathway and, in the case of a suspect VP ECG pattern, an adenosine test could effectively distinguish between the Kent bundle or Mahaim or James fibers (Table 1) [27].

## 4. Electrocardiographic Tips and Clues to Identify a Suspect Accessory Pathway

**Figure 4:**
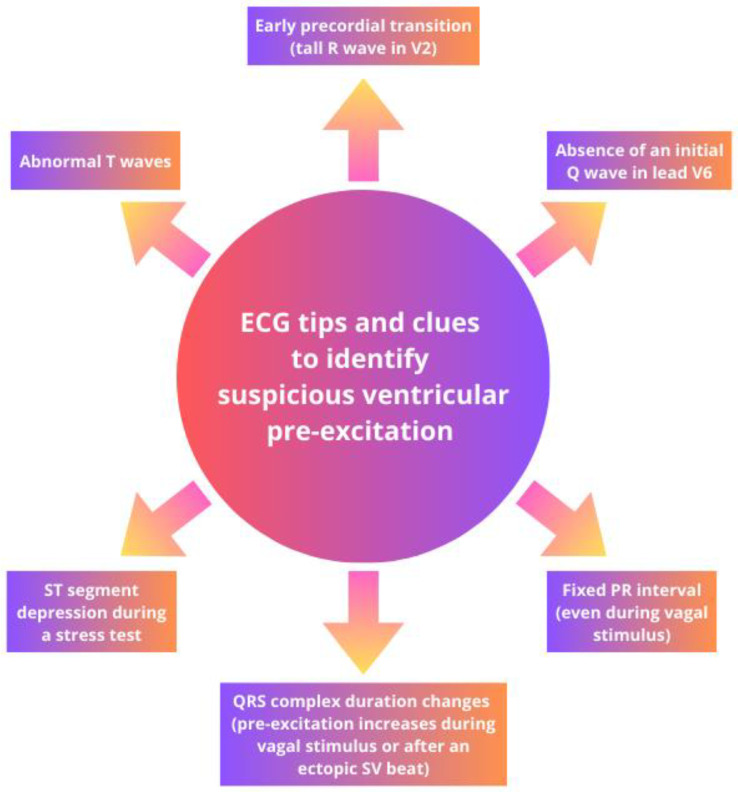
Electrocardiographic tips and clues to identify an accessory pathway in suspicious cases of VP.

In multiple outpatient cases of suspect VP, the knowledge of certain electrocardiographic tips and specific clues can help confirm the diagnosis. These clues can enhance diagnostic sensitivity and allow for the identification of a silent or seemingly hidden accessory pathway. In the presence of suspected Kent-type VP, some simple clues can assist in diagnosis (Figure 4).

These include the following: (1) an early precordial transition, indicated by a tall R wave in V2, and the presence of abnormal T waves; (2) the absence of an initial Q wave in lead V6; (3) a fixed PR interval, even under vagal stimulation; (4) changes in the duration of the QRS complex, with increased VP during vagal stimulation or following an ectopic P wave; and (5) ST-segment depression during exercise testing [28,29]. These elements are useful for accurate and timely diagnosis of VP and could be real “game changers” in everyday clinical practice, especially in sports cardiology outpatient clinics.

**Figure 5:**
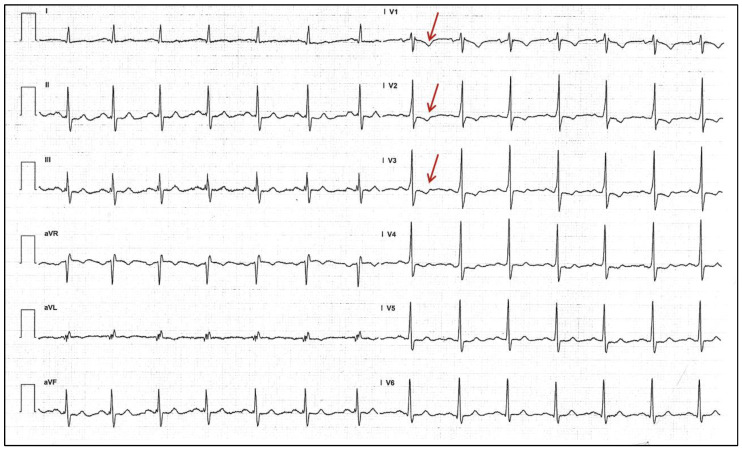
ECG of a patient with ventricular pre-excitation. Focus on the absence of a clear delta wave, while T-wave inversion in V1–V3 leads is visible (red arrows).

Once again, it is important to underline that secondary repolarization abnormalities such as ST-segment depression and T-wave inversion are common and are not indicative of an underlying structural disease (Figure 5). On the contrary, they may be representing a clue for suspecting an AP when the delta wave is not clearly visible.

## 5. What Else Can Be Done When Still in Doubt? The Problem of Latent VP

Latent VP is a condition in which there is an AP between the atria and ventricles that remains electrophysiologically silent under normal circumstances [30]. Unlike overt VP, which is easily detectable on an ECG through the presence of a delta wave, latent VP does not produce any noticeable changes on a resting ECG. It can, however, become apparent during periods of increased atrial rates or through specific diagnostic maneuvers such as vagal maneuvers, pharmacological agents, or atrial pacing. This hidden pathway can potentially contribute to arrhythmias, including atrioventricular reentrant tachycardia (AVRT) and rapid AFib, making its identification and management crucial for preventing arrhythmic events, especially in athletes [1,3,31].

In cases where VP is latent—meaning the accessory pathway is present but not clearly visible on a regular ECG—there are several ways to reveal or “unmask” this hidden pathway [30,32,33]. One approach is using vagal maneuvers, such as the Valsalva maneuver or carotid sinus massage. These techniques stimulate the vagus nerve, which slows down the heart’s conduction through the AVN.

By slowing down the AVN, the AP can become more prominent, making the VP visible. These maneuvers increase vagal tone, slowing conduction through the AVN, and thus favoring the conduction through the AP and making VP more evident on the ECG.

**Figure 6:**
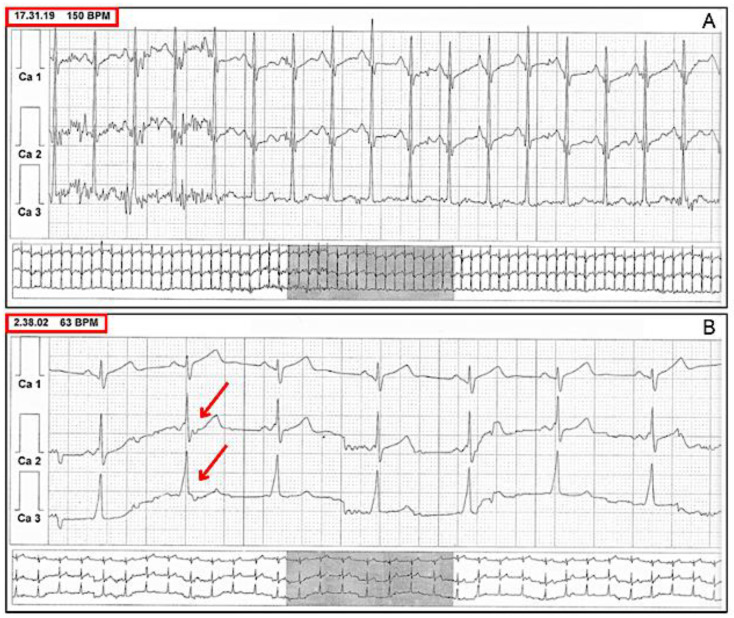
Baseline ECG showing latent and hidden VP during daytime hours (**A**), which becomes apparent during sleep (**B**), unmasked by an increased physiological vagal tone (red arrows indicate enlarged QRS complexes).

Vagal stimulation effects are enhanced during sleep, when the autonomic nervous system shifts towards increased parasympathetic activity and reduced sympathetic activity, giving a rationale for using 24 h ECG monitoring (possibly with a 12-lead configuration) to reveal widening of the QRS during night, importantly with no PR interval prolongation [34] (Figure 6). An exercise stress test is another useful tool, taking advantage of the increase in vagal tone during the recovery phase. Finally, vagal stimulation maneuvers while continuously recording the ECG may also be attempted.

**Figure 7:**
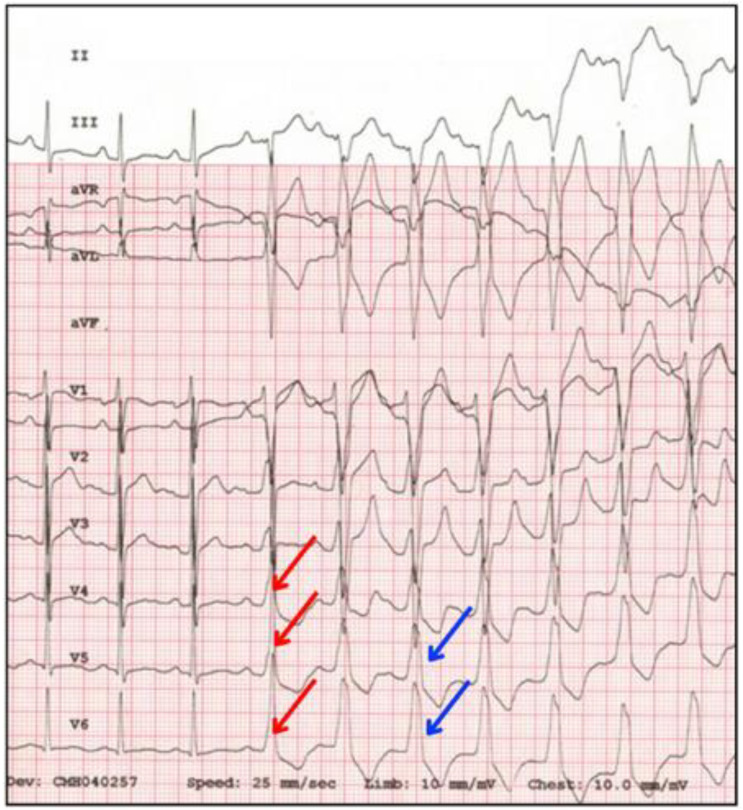
ECG showing the effect of adenosine in a case of latent VP. Following the intravenous administration of the drug, the characteristic delta wave (red arrows) and the wide QRS complex > 120 ms (blue arrows) appear.

Another method involves the use of medications. For instance, adenosine can temporarily block the AV node, which might expose the conduction through the AP (Figure 7). Additionally, antiarrhythmic drugs like procainamide or flecainide can either slow the AV node or directly affect the accessory pathway, making it easier to detect the VP [33,35,36].

**Figure 8:**
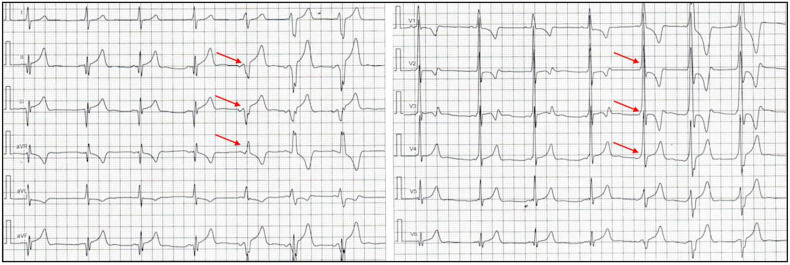
ECG showing the appearance of a delta wave (red arrows) confirming a hidden ventricular pre-excitation that becomes overt after the onset of an ectopic atrial rhythm.

Finally, a useful trick to know in everyday clinical practice is to observe the QRS complex after an atrial ectopic beat. If the origin of the ectopic beat is closer to the AP than the sinus node, or the AVN is still refractory (completely or partially) but the AP is not, the amount of myocardium excited through the AP will increase (Figure 8) [37].

In summary, the interaction between an atrial ectopic beat and an AP can lead to different scenarios. The timing of the beat and the refractory status of the pathway determine whether the impulse is conducted through the pathway, retrogradely, or through the AV node, potentially leading to early ventricular activation or arrhythmias.

## 6. Risk Stratification

In individuals with VP, a proper risk stratification is crucial. Its main intent is to identify those patients at risk for malignant arrhythmic events and SCD. Arrhythmic symptoms were reported by less than half of the individuals who subsequently died, while, in the other half of the cases, SCD represented the first lethal manifestation of the disease [4,28]. As explained before, AP with anterograde and rapid conduction can lead to a very high ventricular rate during AFib, a critical situation with an higher rate of VF degeneration.

Although performance of an electrophysiologic study is recommended to risk stratify individuals with AP, non-invasive evaluation of the AP properties may be useful [2,29,38]. Non-invasive methods include ECG, ambulatory ECG, echocardiography, and exercise stress test at the treadmill.

Rarely, APs may be associated with structural myocardial and valvular diseases, and hence an echocardiography should be performed at least once at the time of diagnosis to rule out some variants of hypertrophic cardiomyopathy and congenital diseases, such as Ebstein anomaly, ventricular non-compaction cardiomyopathy, tricuspid atresia, and congenitally corrected transposition of the great arteries [1,39,40].

Physical stress ECG and 24 h Holter ECG are well known and useful instruments for non-invasive diagnosis [41,42]. VP that becomes intermittent or disappears during exercise implies a high refractory period of the AP even during adrenergic stimulation and is an indication of low-risk AP with a lower rate of malignant events. If the ECG traces are clear-cut (see below), it may be used as an alternative to invasive procedures. However, sudden interruption of VP can be demonstrated only in a minority of cases [41]. Similarly, ambulatory ECG including a training session may demonstrate the disappearance of VP above certain heart rates.

**Figure 9:**
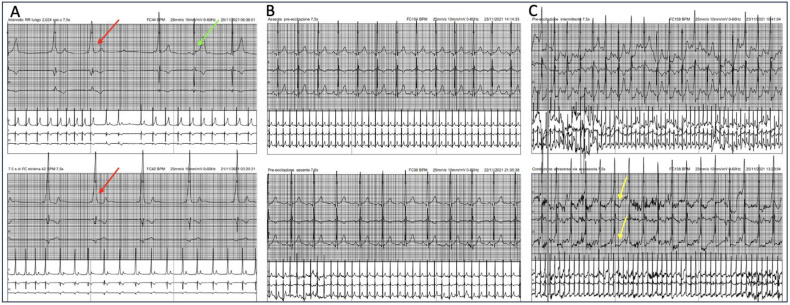
Extracts of 24 h Holter ECG showing intermittent VP: (**A**) intermittent VP at rest, focus on pre-excited beats (red arrows) alternating with non-pre-excited beats (green arrow); (**B**) for heart rate over 80 bpm, VP disappears; (**C**) during training session, at peak effort and maximal heart rate, delta wave and slurred QRS complex re-appear (yellow arrow).

It should be underlined that intermittent VP at rest is not necessarily an indication of a low-risk AP, as adrenergic stimulation can markedly improve the AP refractoriness. Accordingly, in patients with intermittent VP, at least maximal exercise testing and ambulatory monitoring with a training session should be performed to confirm the low-risk AP properties [14] (Figure 9).

When it is impossible to confirm a low-risk AP non-invasively or when multiple APs are suspected, more in-depth invasive assessments need to be carried out. Electrophysiologic study (EPS) is widely intended as the gold standard for these cases [39,43]. Using a specific catheter, the operator can analyze conduction intervals and induce arrhythmias, as supra-ventricular tachycardia (intended as atrioventricular re-entrant tachycardia, AVRT) and AFib, both at baseline and with adrenergic drugs. The most important measure is the anterograde refractoriness of the AP (SPERRI). SPERRI ≤ 250 ms predicts a higher risk of VF and lethal arrhythmic events with a potential ventricular conduction rate of >240 beats per minute, representing an indication for ablation in asymptomatic patients [4,37,43,44,45].

## 7. Stress Test Pitfalls

**Figure 10:**
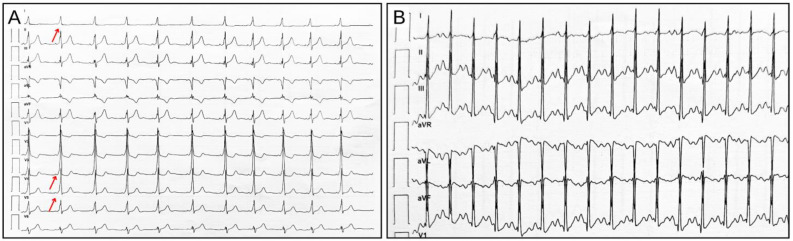
Disappearance of the delta wave during peak effort in the exercise stress test. (**A**) ECG at rest in a patient with a left (mitral) postero-lateral accessory pathway, delta wave, and short PR interval, indicated by the red arrows; (**B**) ECG at peak effort during the exercise stress test: the high heart rate makes it difficult to differentiate between the presence or disappearance of the delta wave.

**Figure 11:**
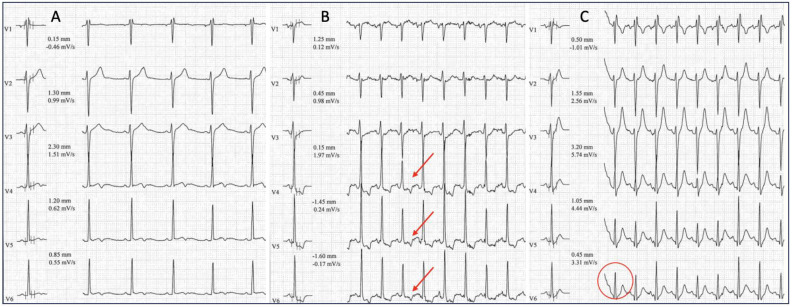
Disappearance of VP during exercise testing (only pre-cordial leads). (**A**) Resting ECG showing delta wave in V3–V6 and secondary repolarization abnormalities. (**B**) During exercise the VP persists, and a ST-segment depression is apparent in V3/4/5/6 (red arrows). ST-segment depression is typical of VP and is not indicative of myocardial ischemia (**C**). Effort peak with disappearance of the delta wave, appearance of a Q wave in V6, and normalization of the ST segment (red circle).

The effort ECG and exercise stress test might conceal some pitfalls that, contrary to the original purpose of the diagnostic tool, could lead to undiagnosed AP or incorrect diagnoses, or could mimic other similar conditions and enhance the risk of arrhythmias. Basically, there are many limitations of interpreting the exercise ECG in athletes with APs, but the two most important are (1) reduction in the AVN conduction times with reduction in the degree of VP (difficult differential diagnosis with disappearance of VP); and (2) depolarization changes mimicking ischemic diseases [7,46]. During physical activity, when the heart rate increases, the delta wave can disappear due to the increase in the conduction time through the AVN connected to a shortened cardiac electrical cycle, although sometimes the delta wave identification can be challenging (Figure 10) [1,15,19]. This could lead to a misinterpretation of the ECG, thereby missing VP diagnosis. Moreover, during physical effort, some patients exhibit intermittent VP with the appearance and disappearance of the delta wave, mimicking other arrhythmic conditions such as multifocal atrial tachycardia or even AFib. ECG changes resembling myocardial ischemia or coronary artery disease manifestations, as ST-segment alterations or T-wave inversion, may also be present and occur following abnormal depolarization and repolarization induced by the AP (Figure 11). These findings may lead to further, and sometimes unnecessary, investigations such as coronary arteriography [46,47]

Drugs used to control heart rate or arrhythmias in patients with VP can also influence the ECG. In a physical effort condition, beta-blockers, for example, can decrease the conduction through the AV node without affecting the AP, leading to exacerbated VP and potential ECG misinterpretation [48,49,50].

## 8. Management of Athletes with VP

Athletes, professional or not, with a VP pattern in the ECG, are exposed to an increased risk of malignant and lethal arrhythmias, although AP is not always altered by physical exercise, regardless of the intensity level [51,52].

**Figure 12:**
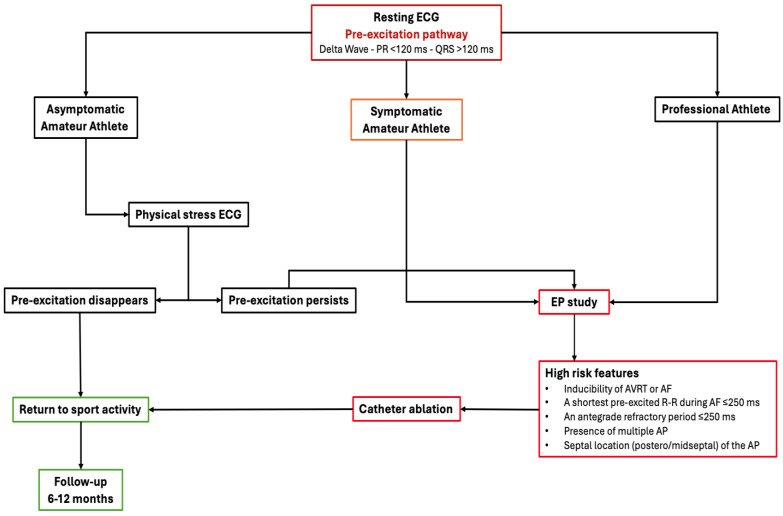
Flow-chart algorithm for diagnostic and therapeutic strategies for athletes with a VP ECG pattern. AF, atrial fibrillation; AP, accessory pathway; AVRT, atrioventricular re-entrant tachycardia; ECG, electrocardiogram; EP, electrophysiological study.

VP can be detected in young asymptomatic athletes through ECG screening, ensuring diagnosis and therapeutic strategies before to avoid the occurrence of major complications, foremost SCD. When this pattern is identified in athletes, risk stratification with electrophysiological study is recommended by both the American Heart Association and the European Society of Cardiology guidelines [2,53,54]. While it is recommended that professional athletes should undergo an EP study in any case, in recreational and amateur athletes with asymptomatic VP, risk assessment and stratification may be pursued by non-invasive testing in the first instance (class IIb indications according to the ESC guidelines). Both guidelines also recommend catheter ablation of high-risk AP or history of AVRT before resuming competitive sports. In normal conditions, athletes can return to exercise as soon as 7–14 days after a successful ablation, but a follow-up ECG should be programmed because late relapses of VP are possible. Furthermore, in case the ablation is not performed because of patient refusal or high procedural risk (e.g., ablation of anteroseptal AP can cause AV block), competitive sport eligibility and participation could be discussed for each single case by taking into consideration the use of pharmacological therapy. In athletes with VP who have not undergone catheter ablation, European guidelines discourage participation, either competitive or recreational, in activities at increased risk (trauma or drowning), in case of the loss of consciousness [38]. An algorithm for risk stratification and management of athletes with VP is shown in Figure 12.

## 9. Conclusions

VP is a heart conduction disorder with a well-defined arrhythmic substrate and specific risk of SCD. While several diagnostic algorithms have been developed during the years, its identification, together with the localization of the AP responsible for VP, represent only the first phase of the diagnostic process. Risk stratification and identification of high-risk patterns should always be part of the complete evaluation of these patients. Further investigations, an exercise stress test, and EP study are crucial for athletes and the general population with high-risk AP, while individuals with low-risk patterns should be managed and followed with specialist non-invasive examinations. Catheter ablation therapy is recommended for high-risk pathways, ensuring the opportunity for a return to professional sport activity after a short period of time. Acknowledgment of the disease and its menacing arrhythmic complications can notably reduce the risk and incidence of SCD, in both professional/amateur athletes and the general population.

## Figures and Tables

**Table 1:** Comparison between different accessory pathways, their localization, and PR interval behavior after adenosine.

Accessory Pathway	Kent Bundle	Mahaim Fibers	James Fibers
Localization	Atrioventricular	Atrio-fascicular Node-ventricular Fascicular-ventricular	Atrio-nodal Atrio-Hisian
Baseline Delta wave	Present	Absent	Absent
Baseline PR interval	Short	Normal	Short
After adenosine PR interval	Unchanged	Increase	Increase

## Data Availability

Not applicable.
